# Dataset of pharmacotherapy in autism spectrum disorder

**DOI:** 10.1016/j.dib.2025.111802

**Published:** 2025-06-18

**Authors:** Sebastião Gonçalves de Barros Neto, Catherine Oliveira de Araújo, Renandro de Carvaho Reis, Kelson James da Silva Almeida, Decio Brunoni, Roberta Monterazzo Cysneiros

**Affiliations:** a Laboratory of Neurobiology, Mackenzie Presbyterian University. Rua da Consolação, 930 – prédio 28, 10 andar – São Paulo- SP, CEP 04048061, Brazil; b Pharmaceutical Science Postgraduate Program. Federal University of Piaui. Teresina – PI, Brazil

**Keywords:** Autism spectrum disorder, Psychopharmaceutical, Medication, Epilepsy, Age

## Abstract

Pharmacological interventions are widely used in autism spectrum disorder (ASD). We investigated the profile of psychopharmaceutical in a sample of children with ASD of low socioeconomic stratum enrolled and the public school in the city of Embu das Artes, Sao Paulo Brazil. From 149 children with a diagnosis of ASD identified in the municipal education department, informant consents were obtained from 129 children's parents. Data collection was performed through clinical evaluation of the children and face to face application of a questionnaire for parents regarding medication use. The participants socioeconomic stratum was mostly C (72.86 %), followed by B (16,27 %), D (10.07 %), and A (0.7 %) who do not undergo specialized multi-professional educational and behavioral intervention. Among the participants 57.36 % were under psychopharmacological intervention and 33.78 % were under a polypharmacy regimen. Typical antipsychotics were used for 35.4 % followed by atypical antipsychotics (27.43 %), antiseizure (19.47 %), and tricyclic antidepressants (7.08 %). The association of risperidone with valproic acid prevailed in 17.68 % of the sample. ASD levels 2 and 3, epilepsy and age were associated with a higher rate of psychopharmaceutical use. The present dataset can be used to investigate the rational use of medication in the ASD population to monitor potential adverse effects, and to compare with the ASD population across the world*.*

Specifications Table

**Table:** 

Subject	Psychiatry and mental health.
Specific subject area	autism spectrum disorder, levels of autism, psychotropic medication survey, the socioeconomic stratum survey*.*
Type of data	Table, demographic data (age, gender, socioeconomic stratum, frequency).
Data collection	The medication usage was collected applying the National Survey on Access, Use and Promotion of the Rational Use of Drugs. The socioeconomic stratum was the determined use a questionnaire – ABEP. Medication was listed and classified using the anatomical therapeutic chemical classification system (ATC)*.*
Data source location	Longitude Oeste: -46.84. Longitude Leste: -46.36. Latitude Norte: -23.36. Latitude Sul: -24.00. City/State: São Paulo/São Paulo Mackenzie Presbyterian University.
Data accessibility	Repository name: Mendeley Data Data identification number: 10.17632/sgvb7sh4yn.5 Direct URL to data: https://data.mendeley.com/datasets/sgvb7sh4yn/5
Related research article	*None.*

## 1 Value of the Data

• This dataset provides information on specific psychotropic medications used by participants, allowing researchers to analyze treatment together with socioeconomic stratum and ASD level.
• Information on psychotropic medications allows comparisons with other studies in which participants receive specialized multi-professional intervention.
• Information on socioeconomic status enables examination of how economic factors may moderate access to medication.
• Future research can utilize this dataset to advance the understanding of the complex interaction among socioeconomic status, ASD level, pharmacological and behavioral interventions.

## 2 Background

Autism spectrum disorder is a developmental disorder marked by social, communication and behavioral deficits [1] Its prevalence has gradually increased over the years ranging from 1 to 150 in 2000, to 1 in 36 in 2020 [2]. This scenario demands more research to develop an effective intervention for this population. In general, individuals with ASD require intensive and comprehensive intervention, based on the principles of applied behavior analysis (ABA) to achieve meaningful outcomes [3]. Psychopharmacological intervention has been widely utilized, ranging from 25% to 65% among children and adolescents with ASD [[4], [5], [6], [7], [8], [9], [10]]. It is applied in specific situations to control symptoms of psychiatric comorbidities and other medical conditions, as epilepsy [[11], [12], [13], [14]]. Polytherapy is common, even in children under of 5 years of age [8,10], which increases the risk of adverse effects [15,16], and places a greater financial burden for families and health systems. Given that most ASD research is conducted in high-income countries, we aimed to provide a dataset of psychotropic medications use in children with ASD from low-income families who do not receive specialized behavioral therapy

## 3 Data Description

Among students enrolled in the public education system of the city of Embu das Artes (SP, Brazil), 140 had a diagnosis of ASD and 129 (92.1 %) participated in the study. Sociodemographic and medical information is provided in Table 1.

**Table 1:** Participants characteristics (n= 129).

Characteristics of the sample	N (%)
Gender	
Male	103 (79.84)
Female	26 (20.16)
Age (mean ± SD)	7.7 ± 3
Age (min-max)	3–17
Severity	
Level 1	46 (35.66)
Level 2	32 (24.81)
Level 3	51 (39.53)
Epilepsy	17 (13.18)
Psychopharmacological intervention	74 (57.36)
Social economic stratum	A (0.7) B (16.27) C (72.86) D/E (10.07)

For the most families, the monthly household income ranged from $ 1965.87 to 3276.76 Brazilian reais (ABEP, 2019), which was 2 or 3 times higher than national minimum wage of $ 998.00 BRL in 2019. Additionally, medications were provided at no cost by the Unified Healthy System (SUS-Sistema Único de Saúde), Brazil´s publicly funded health care system.

Table2 shows the distribution of psychotropic medication use according to participants' gender, ASD level, and the presence of epilepsy. The statistical significance of the distribution was indicated in the table.

**Table 2:** Characteristics of psychopharmaceuticals users and non-users (n= 129).

Characteristics	Use of drugs		χ2 or Fisher’s (p)	OR	CI	LR (*p* value)
	No 55 n (%)	Yes 74 n (%)				
Gender						
Male	44 (80)	59 (79.73)	>0.99	1.266	0.489–3.069	0.664
Female	11 (20)	15 (20.27)		-	-	-
Age	7.20(2.79)	8.12 (3.13)		1.100	0.968–1.251	0.145
Severity						
Level 1	26 (47.27)	20 (27.03)	0.055	1	-	-
Level 2	12 (21.82)	20 (27.03)		2.249	0.884–5.721	0.089
Level 3	17 (30.91)	34 (45.94)		2.375	1.023–5.515	0.044
Epilepsy	5 (9.09)	12 (16.21)	0.297	1.935	0.639–5.860	0.243

Note: χ2 Test. LR: Multivariate analysis by logistic regression. OR: Odds Ratio. CI: Confidence interval.

Table 3 presents the psychotropic medication use among participants, categorized as monotherapy or polytherapy, across gender, ASD severity level and the epilepsy status. The percentage of distribution and statistical significance are indicated in the table.

**Table 3:** Characteristics of individuals on monotherapy and polytherapy (n= 74).

Characteristics	Monotherapy 49 (66.2) n (%)	Polytherapy 25 (33.8) n (%)	χ2 or Fisher’s (p)	OR	CI	LR (*p* value)
Gender						
Male	36 (73.47)	22 (88)	0.2331	4.146	0.866–19.840	0.075
Female	13 (26.53)	3 (12)		-	-	-
Age	7.38 (2.45)	9.56 (3.75)		1.253	1.033–1.521	0.022
Severity						
Level 1	16 (32.65)	4 (16)	0.0248	1	-	-
Level 2	16 (32.65)	4 (16)		1.594	0.299–8.501	0.585
Level 3	17 (34.70)	17 (68)		3.767	0.929–15.278	0.063
Epilepsy	3 (6.12)	9 (36)	0.0019	8.625	2.074–35.865	0.003

Note: LR: Multivariate analysis by logistic regression. OR: Odds Ratio. CI: Confidence interval.

The most prescribed subgroups overall were typical (35.40 %) and atypical (27.43 %) antipsychotics, antiseizure medications (19,47 %), and tricyclic antidepressants (7.08 %). The most prescribed medications were risperidone (24.78 %), periciazine (18,58 %), haloperidol (11,5 %), valproic acid (7,96 %), carbamazepine (7.08 %), and imipramine (5.31%). Regarding polypharmacy regimens, 21 combinations were used, with 56 % of patients taking medications and 44 % taking three or more. The most frequent combination (12%) was the association of valproic acid and risperidone.

## 4 Experimental Design, Materials and Methods

All procedure were approved by the university institutional review board of Mackenzie Presbyterian University (N0. 3.317.217). The sample of this cross-sectional observational study was investigated in 2019, in the public school system in the city of Embu das Artes (SP, Brazil).

In 2019, 24,655 students were enrolled in municipal public schools in the city of Embu das Artes/SP.

One hundred and forty children with a confirmed diagnosis of ASD were identified in the Municipal Education Department and invited to participate in the study. Informed consent was obtained from 129 of children´s parents.

The parents´ interviews were conducted face-to-face, between the months of May and September 2019. The medication usage was collected applying the National Survey on Access, Use, and Promotion of the Rational Use of Drugs [17]. Each medication was listed and classified using the fourth (chemical subgroup) and fifth (chemical/drug) levels of the Anatomical Therapeutic Chemical Classification System (ATC) (World Health Organization, 2019). Participants did not receive payment or other compensation for their time.

The children with confirmed diagnosis of ASD provided by school records were examined by two experts (physician and psychologist) using criteria from Diagnostic and Statistical Manual of Mental Disorders, 5th Edition (DSM-5, APA, 2013) [1] were categorized into different levels of ASD severity: 1 - Mild (requires support), 2 - Moderate (requires substantial support) and 3 – Severe (requires very substantial support). The information obtained also included demographic variables such as children’s age and gender, presence of comorbidities and the parents’ social economic stratum. The socio-economic status was determined using a questionnaire developed by the Brazilian Association of Research Companies (ABEP, 2019).

Continuous variables were expressed as mean and standard deviation and categorical variables as absolute and relative values. To evaluate the frequency of use of psychotropic medication in mono or polytherapy-regime, the following tests were used: the chi-square (χ2) or Fisher’s test and multivariate analysis by logistic regression. Data were analyzed using JASP 0.14.1 software The significance level adopted was (*p*) <0.05 and confidence interval (CI) 95 %.

## Limitations

Of importance, this study has some limitations, among which we mention the relatively small sample size, and the lack of detailed information on the clinical profile, which makes it difficult to relate them to the psychopharmaceuticals prescriptions.

## Ethics Statement

All procedures were approved by the university Institutional Review Board over there Mackenzie Presbyterian University (No. 3.317.217).

## CRediT Author Statement

**Sebastião Gonçalves de Barros Neto:** investigation, data curation, Writing **Catherine Oliveira de Araújo:** investigation, **Renandro de Carvalho Reis:** Formal analysis, **Kelson James Silva de Almeida:** Formal analysis, **Decio Brunoni:** Conceptualization, data curation, Writing - Review & Editing, **Roberta Monterazzo Cysneiros:** Conceptualization, Supervision, Writing - Review & Editing - Original Draft

## Data Availability

Mendeley DataPharmacotherapy in Autism Spectrum Disorder (Original data).
